# Use of nicorandil is Associated with Increased Risk for Gastrointestinal Ulceration and Perforation- A Nationally Representative Population-based study

**DOI:** 10.1038/srep11495

**Published:** 2015-06-29

**Authors:** Chien-Chang Lee, Shy-Shin Chang, Shih-Hao Lee, Yueh-Sheng Chen, Wan-Ting Hsu, Meng-Tse Gabriel Lee

**Affiliations:** 1 Department of Emergency Medicine, National Taiwan University Hospital, Taipei, Taiwan; 2 Department of Emergency Medicine and Department of General Medicine, National Taiwan University Hospital Yunlin Branch, Douliou, Taiwan; 3 Department of Family Medicine, Chang Gung Memorial Hospital, Linkou, Taiwan; 4 Graduate Institute of Clinical Medical Sciences, College of Medicine, Chang Gung University, Taoyuan, Taiwan; 5 Department of Diagnostic Radiology, Kaohsiung Chang Gung Memorial Hospital, Chang Gung University College of Medicine, Kaohsiung, Taiwan

## Abstract

Nicorandil is a vasodilatory drug used to relieve angina symptoms. Several healthcare products regulatory agencies have issued a warning associating the use of nicorandil and gastrointestinal (GI) ulceration. We aimed to evaluate the association between use of nicorandil and GI ulceration/perforation. A population-based cohort study involving 1 million randomly sampled participants in Taiwan’s National Health Insurance Research Database was carried out. We estimated the association between use of nicorandil and GI ulceration/perforation by a Cox proportional hazards regression model. A nicorandil-specific propensity score (PS) was also created for adjustment of 75 covariates and matching. 25.8% (183/710) of nicorandil-treated patients developed new GI ulcer events and 1.6% (20/1254) developed new GI perforation events in the three-year follow-up period, as compared to 9.3% (61,281/659,081) and 0.3% (2,488/770,537) in the general population comparator cohort. Patients treated with nicorandil were at significantly increased risk of GI ulcer (PS adjusted hazard ratio 1.43, 95% CI, 1.23 to 1.65, 6848 excess cases per 100,000 person years) or GI perforation (aHR 1.60, 95% CI 1.02–2.51, 315 excess cases per 100,000 person years) compared with the nicorandil unexposed population. Our finding may warn the clinicians to weigh the overall risk-benefit balance of nicorandil treatment in patients.

Gastrointestinal ulceration or perforation as a potential adverse effect of nicorandil treatment has received much attention recently. Since 1997, there were numerous case report or case series of nicorandil-induced ulcerations in skin and mucous tissue of gastrointestinal tract^1^,^2^,^3^,^4^,^5^,^6^,^7^,^8^,^9^,^10^,^11^,^12^,^13^,^14^,^15^,^16^,^17^,^18^. In almost all of these case reports, the ulcerations were reported to heal upon withdrawal of nicorandil treatment. Thus, several healthcare products regulatory agencies have taken notice of this potential ulceration adverse effect and issued warnings on use of nicorandil.

Nicorandil is a common antianginal medication in Europe and Asia. UK’s 2008 annual prescription data suggested that over 100, 000 people in the U.K. are prescribed with nicorandil^19^. The pharmacological properties of nicorandil came from the nicotinamide ester, which can result in vasodilation of arteries and veins. In several randomized controlled trials, nicorandil has demonstrated equivalent efficacy to nitrate, calcium channel blockers, and beta-blockers in relieving angina symptoms^20^,^21^,^22^,^23^,^24^,^25^,^26^,^27^,^28^,^29^. Unfortunately, these randomized controlled trials did not monitor gastrointestinal (GI) ulceration or perforation as one of the adverse effects.

Since there was no large-scale study conducted to quantify the observed association between nicorandil treatment and GI ulceration/perforation (as far as we were aware), case reports were the only supporting evidence for increased risk of GI ulceration/perforation. Evidence from case reports should be interpreted with caution, due to the limited sample size and the possibility of confounding bias. For example, nicorandil subjects who also took traditional non-steroidal anti-inflammatory drugs were predisposed to 3 fold higher risk of GI ulceration or perforation^30^,^31^,^32^. Thus, there is a need to correct for the known risk factors for GI ulceration or perforation, before the association between nicorandil and GI ulceration/perforation can be suggested. With the limitation of the prior studies in mind, we used a 1 million national representative cohort to study the potential link between nicorandil treatment and risk of GI ulceration/perforation.

## Methods

### Setting and Data Collection

We carried out a population-based cohort study using the National Health Insurance Research Database (NHIRD) of Taiwan, done in accordance with STROBE guideline and under the approval of the institutional review board of National Taiwan University Hospital. The database contains de-identified secondary data, and met the requirements of the “Personal Information Protection Act” in Taiwan. Thus, the data were analyzed anonymously and the need for informed consent was waived.

Several studies have showed that the NHIRD is appropriate for use in pharmacoepidemiologic research^33^,^34^,^35^. The demographics and complete claim history of 1 million representative Taiwanese can be found in the NHIRD database. Detailed claim history includes electronic claim records of outpatients, inpatients, pharmacy prescription, quantity of medications, route of administration, diagnoses, operations, and procedures.

### Study population

We used a study cohort of NHIRD that consists of a longitudinally followed up Taiwanese population from January 2005 to December 2009. All participants in the NHIRD who were aged 20 years and over at 1 January 2005 and had at least one inpatient or outpatient visit in the previous 6 months were eligible for inclusion. Considering the time-varying risk after initial exposure to nicorandil, we adopted a new user cohort design^36^, in which previous users of nicorandil were excluded before cohort entry. We excluded all patients who received at least one prescription of nicorandil in 2005, and assessed the nicorandil exposure status in 2006. Nicorandil users were defined as subjects who redeemed one or more prescriptions for nicorandil. In Taiwan, the prescription length of nicorandil is usually between 14 and 28 days. Patients entered the cohort on the first day of year 2007 and were followed up for 3 years until the first occurrence of the either event: diagnosis of gastrointestinal ulcer/ perforation, termination of health insurance coverage, death, or end of the study period, whichever came first. We do not aim to study the risk of GI ulceration/perforation recurrence; therefore, patients who had been diagnosed with GI ulceration or perforation before 2007 were excluded for analysis. Considering GI ulceration and GI perforation are two related but different diseases, we created two separate cohorts for analysis. Cohort 1 excluded prevalent cases of GI ulceration before 2007 and cohort 2 excluded prevalent cases of GI perforation before 2007. In addition, patients with the following special conditions that can lead to increase risk in GI ulceration/ perforation were excluded: ingestion of corrosives (ICD-9 CM 983), Behcet’s syndrome (ICD-9 CM 136.1), disorders involving the immune mechanism (ICD-9 CM 279), polyarteritis nodosa and allied conditions (ICD-9 CM 446), diffuse diseases of connective tissue (ICD-9 CM 710), rheumatoid arthritis and other inflammatory polyarthropathies (ICD-9 CM 714).

### Outcomes

Cohort 1 was assessed for GI ulceration and cohort 2 was assessed for GI perforation. GI ulceration was defined by ICD9-CM codes associated with either upper GI ulceration (530.2, 531.X, 532.X, 533,X, 534.X) or lower GI ulceration (569.41 and 569.82) plus procedure code for esophagogastroduodenoscopy or colonoscopy. GI perforation was defined by ICD9-CM with either one of the following type of perforation: gastric perforation (531.1, 531.2, 531.5, 531.6, 532.1, 532.2, 532.5, 532.6, 533.1, 533.2, 533.5, 533.6, 534.1, 534.2, 534.5, 534.6) and small or large intestinal perforation (569.83) plus procedure code for laparotomy or computed tomography. NHIRD prevents the linkage between claim database and medical records; therefore we could not perform the validation of outcome in these 2 cohorts. Instead, we tested the accuracy of our outcome definition by performing an independent validation on one hundred electronic medical records from a university hospital. The combined diagnostic and procedure code definition in this study have a positive predictive rate of 83% for GI ulceration and 89% for GI perforation.

### Covariates

In order to be as comprehensive as possible in adjusting for factors that might confound the drug-outcome association, we reviewed literature for covariates related to gastrointestinal ulceration/perforation and angina (the main indication for nicorandil). 75 relevant covariates (Table 1) were chose and assessed from January 2005 to December 2005. There are seven category of covariates: demographic variables, risk factors for intestinal ulceration/perforation, respiratory comorbidities, cardiovascular comorbidities, musculoskeletal comorbidities, healthcare service utilization, and use of specific medications. Each individual’s burden of comorbidity was quantified by a combined weighted comorbidity score. This index is an improved comorbidity index based on the Elixhauser system^37^. The score contains common comorbidities such as myocardial infarction, congestive heart failure, peripheral vascular disease, cerebrovascular disease, dementia, chronic pulmonary disease, connective tissue disease, ulcer disease, liver disease, diabetes, hemiplegia, renal disease, neoplasms and AIDS.

### Data Analysis

The baseline characteristics of participants were described and compared among nicorandil users and nonusers (Table 1). To examine differences in baseline characteristics between nicorandil users and nonusers, we used Pearson chi-square tests for comparison of dichotomous variables and Mann-Whitney U tests for continuous variables. For multivariate analysis, we constructed Cox proportional hazards models to derive hazard ratios and 95% confidence intervals. We tested the proportional hazards assumption by introducing an interaction term of exposure and follow-up time in the model. In addition, we confirmed the assumption of proportional hazards by an examination of the log (minus log) curves (appendix 1).

To consolidate the strength of our findings, we calculated the hazard ratios by three different methods. The first method obtains an unadjusted crude estimate. The second method obtains an adjusted effect estimate by entering the propensity score (PS) into the Cox regression model as a continuous covariate plus a quadratic term to allow nonlinearity. The PS was built by a multivariate logistic model using the prescription of nicorandil as the dependent variable, and the 75 covariates as the independent variables. The PS model (appendix 2, 3) showed high predictability (c- statistic: 0.91, appendix 4) of nicorandil prescription. Finally, using the COX-model, we carried out stratifying on the matched pairs. The matching was done using the greedy matching algorithm without any trimming^38^. We examined the distribution of PS in the study population and checked the balance of each covariate after PS matching by using absolute standardized difference (appendix 5). Standardized differences between the two treatment groups were calculated as the differences in the either the means or the percentage, divided by the pooled standard deviation.

To assess the robustness of the hazard ratios for risk of GI ulceration/perforation in relation to the duration of follow-up, we carried out a subgroup analysis. Predefined subgroups included sex and age of 75 years. To find out if nicorandil users and PS-matched non-users have different cumulative hazard of GI ulceration/perforation, we used the Nelson-Aalen estimators to generate a cumulative hazard function and plot it over time. All analyses were carried out with SAS 9.3 for Windows (SAS Institute Inc, Cary, NC) and the data are reported in accordance with STROBE guideline.

## Results

### Participant Enrollment and Baseline Characteristics

The baseline characteristics of the two cohorts were displayed on Table 1. The source population comprises of 1 million participants with 3 years of follow-up. After exclusion of existing users of nicorandil and prevalent cases of GI ulceration/perforation in the pre-enrollment period, there were 710 nicorandil users in the GI ulceration cohort and 1,254 nicorandil users in the GI perforation cohort. In both cohorts, there were significant differences in the baseline characteristics between nicorandil users and nonusers. Users of nicorandil represented a group of patients with older age, more urban and suburban residents, higher burden of comorbidity, and used more anti-inflammatory, cardiovascular, and antipsychotics medications. We used PS for matching nicorandil users and nonusers. After matching, there were negligible standardized differences in the baseline covariates between nicorandil users and nonusers (appendix 5).

### Outcome- GI ulceration

To investigate whether use of nicorandil has differential effects on different part of the GI system, we classified the location of GI ulceration as either upper GI (esophagus, stomach, and small intestine) or lower GI ulceration (large intestine and anus). There are more outcomes of upper GI ulceration outcome (N =181) as compared to lower GI ulceration (N = 2). (Table 2).

During a 3 year follow-up period, nicorandil users were found to have higher incidence of upper GI ulceration (25.5%) as compared to nonusers (9.1%) and propensity score (PS) matched nonusers (18.9%). However, there was no significant difference in the incidence of lower GI ulceration (0.3%) among nicorandil users as compared to nonusers (0.2%).

Unadjusted analysis showed that nicorandil therapy was associated with an increased risk of overall GI ulceration (HR 3.10; 95%CI, 2.68-3.59). (Table 3) After adjusting for potential confounders using the PS in the Cox model, nicorandil therapy was still significantly associated with GI ulceration (HR 1.43; 95%CI, 1.23-1.65). PS-matched analysis yielded a similar effect size (HR 1.41, 95%CI 1.13-1.76). The adverse effects of nicorandil therapy were found in the upper GI tract but not in the lower GI tract.

### Outcome- GI perforation

The association between nicorandil therapy and GI perforation is summarized in Tables 2 and 3. GI perforation was classified as either upper GI (gastric) perforation or lower GI (small or large intestinal) perforation. There are more outcomes of upper GI perforation (N = 19) as compared to lower GI perforation (N = 1).

The crude incidence of upper GI perforation was higher in nicorandil users (1.5%) as compared to nonusers (0.2%) and PS matched non-users (1.2%). There was no significant difference in the crude incidence of lower GI perforation between nicorandil users (0.08%) and PS matched non-users (0.08%).

Unadjusted analysis showed that nicorandil therapy was associated with an increased risk of overall GI perforation (HR, 3.82; 95%CI, 2.46–5.93). PS adjustment decreased the effect size (HR, 1.60, 1.02–2.51), and PS matching further attenuates the effect (HR 1.25, 95% 0.65–2.42). The effect estimates associated with the upper gastric perforation was very similar to the effect estimates obtained for overall GI perforation. Although the effect was not significant, we detected a 68% higher risk of lower GI perforation among the nicorandil treated anginal patients (PS-adjusted HR 1.68, 0.23–12.3).

### Time-varying risk analysis

**Figure 1:**
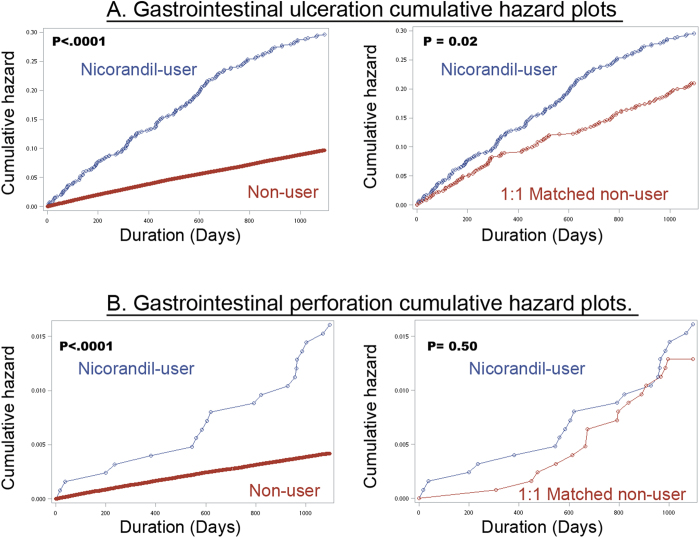
Cumulative hazard plots for gastrointestinal ulceration (**A**) and gastrointestinal perforation (**B**). All the left panels display the combined cumulative hazard of all the nicorandil non-users. In the right panels, only the cumulative hazard of the propensity score matched nicorandil non-users are displayed. The p-values were calculated by log- rank test.

To describe the time-varying nature of nicorandil associated ulceration/perforation risk, we draw a hazard function plot over the three year follow-up period (Fig. 1). We found that the cumulative hazard increased at a faster rate for nicorandil users than nonusers for both the GI ulceration (Fig. 1A) and GI perforation (Fig. 1B) events. There was no apparent sign of the cumulative hazard plateauing for both nicorandil users and nonusers for both the GI ulcer/perforation cohort. To remove the confounding effect, we further plotted the cumulative hazard over time for 1:1 PS-matched cohort (Fig. 1 right panel). For GI ulceration, the cumulative hazard is consistently higher than PS matched nonusers. For GI perforation, the cumulative hazard was higher for nicorandil users in the first 800 days after exposure, and there is a trend toward undifferentiated risk between users and PS matched nonusers after 800 days (Fig. 1B right panel).

### Subgroup analysis

To investigate whether there is a differential risk among different populations, we performed analyses on pre-defined age and gender subgroups (Table 4). The interaction term did not reach statistical significance (p-value < 0.05) for any of the subgroups. Compared with non-nicorandil user, the association between GI ulceration/perforation and use of nicorandil was consistent across the subgroups within their cohort. The only exception is the >75 years of age subgroup in the GI perforation cohort, which has a lower risk of GI perforation.

## Discussion

We carried out a population-based study involving one million national representative participants. After adjustment for PS, use of nicorandil was associated with a 1.4 fold increase in risk for GI ulceration and 1.6 fold increase in risk for GI perforation. The risk of for GI ulceration was consistently higher in the entire three year follow-up period, but risk for GI perforation seemed to be higher only in the first 800 days after exposure.

To the best of our knowledge, the risk of GI ulceration/perforation among nicorandil users has not previously been examined in a large general population. Several randomized controlled trials (RCT) have shown nicorandil to be an effective and safe drug in relieving angina symptoms^20^,^21^,^22^,^23^,^24^,^25^,^26^,^27^,^28^,^29^,^39^. These RCTs, however, did not associate mucosal and cutaneous ulcerations with nicorandil treatment. The adverse effects of GI ulceration/perforation may be undetected in RCTs if ulceration was not listed as one of the actively surveyed safety endpoints. In addition, most RCTs have a small sample size and exclude elderly patients with multiple comorbidities^40^,^41^. It was not until the first case report in 1997 that nicorandil treatment was suspected to cause oral ulcers^1^,^2^. Since then, many case reports of nicorandil induced GI ulcerations and GI perforations associated with elderly patients have been published^11^,^12^,^15^,^42^,^43^,^44^. Although the increased incidence of GI ulceration/perforation in patients treated with nicorandil must be validated in other cohorts, evidences from existing clinical observation provide substantial support for our result.

Currently, the best biological hypothesis on how nicorandil might induce ulceration comes from a single patient study that showed ulceration might result from the increased concentrations of nicorandil metabolites in the edge of a previously injured area^45^. Since it is extremely difficult to conduct any large scale clinical studies involving injured subjects with use of nicorandil, it is hoped that scientists can use the animal model to decipher the biological mechanism of nicorandil induced GI ulcerations or GI perforation.

Despite the significant risk of nicorandil associated GI ulceration/perforation identified in this study, clinical decision on nicorandil treatment should consider the background incidence of GI ulceration and GI perforation in a similar (PS-matched) population. In this cohort, the observed risk in the nicorandil treated cohort corresponded to 6848 excess cases of GI ulceration and 315 excess cases of GI perforation per 100,000 person years. In other words, if the observed association were causal, there will be one additional case of nicorandil induced GI ulceration in every 15 nicorandil users, and one additional case of nicorandil induced GI perforation in every 317 nicorandil users. Given the high frequency of nicorandil induced GI ulceration and the high mortality associated with GI perforation, physicians should really weigh the overall risk-benefit balance of nicorandil treatment in patients at high risk for GI ulceration/perforation.

Results of study should be interpreted in light of both strengths and limitations. The use of a national representative database ensured minimal risk of selective population and related potential bias. In addition, excluding all existing users and cases from analysis may help to minimize the survivor bias. We constructed a highly discriminative PS (C-statistic, 0.91) and use it for matching users and nonusers, which we believe may greatly alleviate confounding by indication. Confounding by indication may have arisen if nicorandil was prescribed for patients at increased risk for GI ulceration/perforation.

Our study also bears some limitations. First, even though we tried to be as comprehensive as possible in adjusting/matching baseline characteristics, there will always be unmeasured confounders. Since we were studying on a claims database, many life style factors such as alcohol drinking and smoking are missing. Both of these factors pose an increase in risk to GI ulceration and GI perforation^46^,^47^,^48^,^49^,^50^. We used alcohol- or smoking-related diseases as a proxy for confounding adjustment; nevertheless, we cannot totally exclude the possibility of residual confounding. Secondly, we also cannot rule out the possibility of exposure misclassification. The claims database had no record on whether nicorandil was actually taken by patients. Non-compliance with nicorandil could result in misclassification of non-users to users, but could not misclassified users to non-users. Finally, an even larger study population will be required for answering whether use of nicorandil has differential effect on different parts of the GI system.

This study based on more than 600,000 randomly selected patients found a 43% increase in risk of GI ulceration and a 60% increase in the risk of GI perforation in nicorandil treated patients. The augmented risk of GI complications adds significantly to existing evidence. Given the high mortality and morbidity associated with GI complications, these finding may warn the clinicians to weigh the overall risk-benefit balance of nicorandil treatment in patients at high risk for GI ulceration or perforation.

## Additional Information

**How to cite this article**: Lee, C.-C. *et al.* Use of nicorandil is Associated with Increased Risk for Gastrointestinal Ulceration and Perforation- A Nationally Representative Population-based study. *Sci. Rep.* **5**, 11495; doi: 10.1038/srep11495 (2015).

## Supplementary Material

Supplementary Information

## Figures and Tables

**Table 1:** Participant Enrollment and Baseline Characteristics.

	Cohort 1: GI ulcer			Cohort 2: GI perforation		
	Nicorandil User(N = 710)	Non-user (N = 659,081)	P-value	Nicorandil User(N = 1254)	Non-user(N=770,537)	P-value
Demographics						
Gender male (%)	412 (58.0)	345387 (52.4)	0.003	703 (56.1)	397156 (51.5)	0.0014
Age	65.1 ± 12.3	44.3 ± 17.4	<.0001	65.9 ± 12.2	45.1 ± 17.5	<.0001
Area						
Urban Area	212 (29.9)	202929 (31.0)	<.02	357 (28.6)	236290 (30.9)	0.0002
Metro Area	195 (27.5)	189394 (28.9)		346 (27.7)	222360 (29.1)	
Suburban Area	220 (31.1)	207883 (31.8)		400 (32.0)	242374 (31.7)	
Countryside Area	81 (11.4)	54300 (8.3)		147 (11.8)	64496 (8.4)	
Insurance premium level						
Dependent	55 (7.8)	33719 (5.1)	<.001	106 (8.5)	39484 (5.1)	<.0001
<666 USD	211 (29.7)	164281 (24.9)		391 (31.2)	185753 (24.1)	
666-1331 USD	308 (43.4)	292151 (44.3)		557 (44.4)	345439 (44.8)	
>= 1331 USD	136 (19.2)	168930 (25.6)		200 (16.0)	199861 (25.9)	
Comorbidity score						
Comorbidity score	0.52 ± 1.42	0.1 ± 0.57	<.0001	0.65 ± 1.47	0.13 ± 0.65	<.0001
Baseline comorbidities						
Diabetes	202 (28.5)	30904 (4.7)	<.0001	381 (28.5)	43487 (5.6)	<.0001
Disease related to use of alcohol	14 (1.9)	4736 (0.7)	<.0001	23 (1.8)	6532 (0.9)	<.0001
Disease related to use of tobacco	8 (1.1)	2948 (0.5)	0.007	13 (1.0)	3930 (0.5)	0.007
Psychiatric disorder	141 (19.9)	40667 (6.2)	<.0001	329 (26.2)	60376 (7.8)	<.0001
Neurologic disorder and spinal cord injury	28 (3.9)	5537 (0.8)	<.0001	58 (4.6)	7814 (1.0)	<.0001
Immunocompromised states	64 (9.0)	13871 (2.1)	<.0001	128 (10.2)	20766 (2.7)	<.0001
Cancer (excluding GI cancer)	32 (4.5)	9869 (1.5)	<.0001	66 (5.3)	14818 (1.9)	<.0001
Congenital renal disease and acquired renal disease	37 (5.2)	5873 (0.9)	<.0001	92 (7.3)	8888 (1.2)	<.0001
Renal failure and hemodialysis	42 (5.9)	3619 (0.6)	<.0001	93 (7.4)	5717 (0.7)	<.0001
Benign prostatic hyperplasia	87 (12.3)	9034 (1.4)	<.0001	177 (14.1)	14333 (1.9)	<.0001
Anemia	27 (3.8)	9180 (1.4)	<.0001	69 (5.5)	14044 (1.8)	<.0001
Bed-ridden status	11 (1.6)	3538 (0.5)	<.0001	25 (1.9)	5030 (0.6)	<.0001
Aortic dissection and aortic aneurysm	0	197 (0.0)	0.64	1 (0.1)	279 (0.1)	0.64
Obesity, diagnosed, not morbid	5 (0.7)	1356 (0.2)	<.0001	6 (0.5)	1733 (0.2)	<.0001
Malnutrition and postgastric surgery	6 (0.9)	1298 (0.2)	<.0001	14 (1.1)	2249 (0.3)	<.0001
Amputation	0	46 (0.02)	<.0001	0	65 (0.01)	<.0001
Chronic liver disease and cirrhosis	86 (12.1)	28279 (4.3)	<.0001	179 (14.3)	43335 (5.6)	<.0001
Organ transplant	0	140 (0.0)	0.69	1 (0.1)	196 (0.0)	0.22
Serious neuromuscular	3	499 (0.1)	0.001	4 (0.3)	688 (0.1)	0.006
Gastrointestinal Risk factors						
appendicitis	1 (0.1)	878 (0.1)	0.96	23 (1.8)	6532 (0.9)	0.41
Colorectal cancer	8 (1.1)	1381 (0.2)	<.0001	13 (1.0)	3930 (0.5)	<.0001
Esophageal cancer	0	96 (0.0)	0.74	329 (26.2)	60376 (7.8)	0.19
Stomach cancer (also called gastric cancer)	2 (0.3)	282 (0.0)	0.0022	58 (4.6)	7814 (1.0)	<.0001
Inflammatory Bowel Disease (chronic)	6 (0.9)	3478 (0.5)	0.24	128 (10.2)	20766 (2.7)	<.0001
Ulcerative Enterocolitis	1 (0.1)	237 (0.0)	0.14	66 (5.3)	14818 (1.9)	0.62
superior mesenteric artery syndrome	0	6 (0.0)	0.93	92 (7.3)	8888 (1.2)	0.89
trauma (as exclusion for the intestinal perforation at the same time )	58 (8.2)	35034 (5.3)	0.0007	93 (7.4)	5717 (0.7)	<.0001
Crushing Injury	4 (0.6)	2807 (0.4)	0.57	177 (14.1)	14333 (1.9)	0.03
ascariasis	0	108 (0.0)	0.73	69 (5.5)	14044 (1.8)	0.12
Typhoid fever (acute)	0	23 (0.0)	0.87	25 (1.9)	5030 (0.6)	0.83
Respiratory comorbidities						
Chronic obstructive pulmonary disease (COPD)	99 (13.9)	16225 (2.5)	<.0001	217 (17.3)	24935 (3.2)	<.0001
Asthma	56 (7.9)	12617 (1.9)	<.0001	115 (9.2)	18204 (2.4)	<.0001
Pulmonary heart disease	3 (0.4)	308 (0.1)	<.0001	7 (0.6)	480 (0.1)	<.0001
Cardiovascular comorbidities						
Congestive heart failure	96 (13.5)	6077 (0.9)	<.0001	180 (14.4)	9242 (1.2)	<.0001
Cerebrovascular disease	83 (11.7)	10477 (1.6)	<.0001	158 (12.6)	15535 (2.0)	<.0001
Myocardial infarction/acute coronary syndromes	22 (3.1)	1087 (0.2)	<.0001	45 (3.6)	15588 (0.2)	<.0001
Stroke or transient ischemic attack	39 (5.5)	5017 (0.8)	<.0001	72 (5.7)	7501 (0.9)	<.0001
Peripheral arterial disease	16 (2.3)	2075 (0.3)	<.0001	27 (2.2)	3180 (0.4)	<.0001
Angina	84 (11.8)	4592 (0.7)	<.0001	180 (14.4)	7343 (0.9)	<.0001
Other ischemic heart disease	238 (33.5)	14647 (2.2)	<.0001	450 (35.9)	22766 (2.9)	<.0001
Cerebral atherosclerosis	11 (1.6)	1041 (0.2)	<.0001	19 (1.5)	1674 (0.2)	<.0001
Cardiac valve disease	29 (4.1)	4924 (0.8)	<.0001	67 (5.3)	7236 (0.9)	<.0001
Conduction disorder	5 (0.7)	337 (0.1)	<.0001	8 (0.6)	521 (0.1)	<.0001
Arrhythmia	89 (12.5)	10001 (1.5)	<.0001	183 (14.6)	14955 (1.9)	<.0001
Hypertension	349 (49.2)	56224 (8.5)	<.0001	660 (52.6)	78657 (10.2)	<.0001
Hyperlipidemia	173 (24.4)	31328 (4.8)	<.0001	337 (26.9)	44319 (5.8)	<.0001
CV congenital anomalies (CA)	0	35 (0.01)	0.84	1 (0.1)	44 (0.0)	<.0001
Baseline musculoskeletal comorbidities						
Ankylosing spondylitis	8 (1.1)	1931 (0.3)	<.0001	13 (1.0)	2774 (0.4)	<.0001
Congenital musculoskeletal anomalies	0	1	0.97	0	1	0.97
Gouty arthritis	91 (12.8)	19500 (2.9)	<.0001	173 (13.8)	27022 (3.5)	<.0001
Arthropathy associated with systemic disorders	253 (35.6)	66198 (10.0)	<.0001	502 (40.0)	94417 (12.3)	<.0001
Healthcare Service Utilization						
Number of OPD visit	30.7 ± 22.2	11.5 ± 13.8	<.0001	30.6 ± 22.1	11.5 ± 13.8	<.0001
Number of emergency department visit	0.42 ± 1.10	0.11 ± 0.52	<.0001	0.42 ± 1.13	0.11 ± 0.52	<.0001
Number of hospitalization	0.47 ± 1.06	0.11 ± 0.51	<.0001	0.48 ± 1.06	0.11 ± 0.51	<.0001
Medication						
NSAIDs	319 (44.9)	125692 (19.1)	<.0001	629 (50.2)	169687 (22.0)	<.0001
Aspirin	295 (41.6)	25270 (3.8)	<.0001	523 (41.7)	36536 (4.7)	<.0001
Systemic immunosuppressive agents and biologics	0	422 (0.06)	0.50	1 (0.1)	579 (0.1)	0.95
Systemic corticosteroids	113 (15.9)	34786 (5.3)	<.0001	219 (17.5)	48200 (6.3)	<.0001
DMARDs	4 (0.6)	4774 (0.7)	0.61	10 (0.8)	6122 (0.8)	0.99
Statin	131 (18.5)	15719 (2.4)	<.0001	260 (20.7)	22385 (2.9)	<.0001
ACE inhibitors	146 (20.6)	20466 (3.1)	<.0001	276 (22.0)	28890 (3.8)	<.0001
Oral hypoglycemic	156 (21.9)	23220 (3.5)	<.0001	309 (24.6)	32074 (4.2)	<.0001
Antipsychotic	81 (11.4)	19433 (2.9)	<.0001	205 (16.4)	29368 (3.8)	<.0001
Antidepressants	1 (0.1)	1890 (0.3)	0.46	6 (0.5)	2439 (0.3)	0.31

**Table 2:** Gastrointestinal ulceration and perforation outcomes in users and nonusers of nicorandil.

Gastrointestinal ulceration			
	Nicorandil User (N = 710)	Non-user (N = 659,081)	Matched Non-user (N = 708)
Ulceration of esophagus, stomach and small intestine	181 (25.5%)	60,126 (9.1%)	134 (18.9%)
Ulceration of large intestine and anus	2 (0.3%)	1,155 (0.2%)	0*
**Gastrointestinal perforation**			
	Nicorandil User (N=1,254)	Non-user (N=770,537)	Matched Non-user (N=1250)
Gastric perforation	19 (1.5%)	1,235 (0.2%)	15 (1.2%)
Small or large intestinal perforation	1 (0.08%)	1,253 (0.2%)	1 (0.08%)

^*^In the matched cohort, we were unable to find a non-user with ulceration of large intestine and anus.

**Table 3:** Crude and adjusted effect measure for the association between use of Nicorandil and risk of incident gastrointestinal ulcer and perforation.

	Crude effect estimate (HR, 95% confidence interval)	Propensity score adjusted (HR, 95% confidence interval)	Propensity score matched (HR, 95% confidence interval)
Overall gastrointestinal ulceration	3.10 (2.68–3.59)***	1.43 (1.23–1.65)***	1.41 (1.13–1.76)***
Ulceration of esophagus, stomach and small intestine	3.13 (2.70–3.62)***	1.43 (1.23–1.66)***	1.40 (1.12–1.75)***
Ulceration of large intestine and anus	1.31 (0.18–9.28)	1.11 (0.15–8.00)	NA
Overall gastrointestinal perforation	3.82 (2.46–5.93)***	1.60 (1.02–2.51)*	1.25 (0.65–2.42)
Gastric perforation	3.78 (2.41–5.93)***	1.61 (1.02–2.55)*	1.27 (0.65–2.50)
Small or large intestinal perforation	6.22 (0.87–44.6)	1.68 (0.23–12.3)	1.00 (0.06–16.0)

HR refers to hazard ratio

*refers to p<0.05, **refers to refers to p < 0.01, and ***refers to refers to p < 0.001.

**Table 4:** Effect of Nicorandil participant subgroups on risk of gastrointestinal ulcer and perforation.

	Patient subgroups	Propensity score adjusted HR (95% Confidence interval)	Interaction term P-value
Gastrointestinal ulcer	>75 years of age	1.54 (1.12–2.12)**	0.42
	<=75 years of age	1.42 (1.20–1.67)***	
	Male	1.37 (1.11–1.68)**	0.40
	Female	1.46 (1.18–1.80)***	
Gastrointestinal perforation	>75 years of age	1.19 (0.44–3.21)	0.34
	<=75 years of age	1.78 (1.08–2.94)*	
	Male	1.66 (0.91–3.03)	0.91
	Female	1.59 (0.82–3.10)	

HR refers to hazard ratio. *refers to p < 0.05, **refers to refers to p < 0.01, and ***refers to refers to p < 0.001.

## References

[b1] Boulinguez S. *et al.* [Giant buccal aphthosis caused by nicorandil]. Presse medicale 26, 558 (1997). 9161430

[b2] Reichert S. *et al.* Major aphthous stomatitis induced by nicorandil. European Journal of Dermatology 7, 132–133 (2000).

[b3] Salim F., Joshi A. & Hopkins C. Ulceration of the nasal dorsum: a rare cause? J. Laryngol. Otol. 128, 289–291, 10.1017/S0022215113003629 (2014). 24472676

[b4] Robinson A., Baker P. & Stevenson H. Nicorandil as a cause of perineal ulceration. Ulster Med. J. 81, 97 (2012). 23526854 PMC3605543

[b5] Shams K., Mukkanna K. S., Loney M. D., Evans C. D. & Shaffrali F. C. Leg ulcers associated with nicorandil are possibly underdiagnosed. Clin. Exp. Dermatol. 38, 193–194, 10.1111/j.1365-2230.2012.04475.x (2013). 23083409

[b6] Mikeljevic J. & Highet A. S. Nicorandil-induced leg ulceration without mucosal involvement. Clin. Exp. Dermatol. 36, 372–373, 10.1111/j.1365-2230.2010.03932.x (2011). 21564175

[b7] Yap T. *et al.* Nicorandil-induced penile ulcerations: a case series. BJU Int. 107, 268–271, 10.1111/j.1464-410X.2010.09463.x (2011). 20575979

[b8] Chan S. K., Harris M. D., Baldwin P. J. & Sterling J. C. Vulvovaginal ulceration during prolonged treatment with nicorandil. BJOG 116, 1403–1405, 10.1111/j.1471-0528.2009.02259.x (2009). 19538411

[b9] Fraser S. J., Pinion S. B., Adamson B. & Allan S. J Vulval ulceration induced by the potassium-channel activator Nicorandil: a case series of five patients. BJOG 116, 1400–1402, 10.1111/j.1471-0528.2009.02260.x (2009). 19538410

[b10] Bhatti I., Cohen S. N., Bleiker T., Lund J. & Tierney G. Nicorandil-induced foreskin ulceration. Colorectal Dis. 11, 424–425, 10.1111/j.1463-1318.2008.01661.x (2009). 18684152

[b11] Lee B. C., Allen P. B., Caddy G. R. & Mainie I. Nicorandil associated colonic ulceration: case series of an increasingly recognized complication. Dig. Dis. Sci. 56, 2404–2408, 10.1007/s10620-011-1634-x (2011). 21380762

[b12] Malde S. & Wilson A. Rectal ulceration caused by the anti-anginal nicorandil: Case report of a preventable complication. Patient Saf. Surg. 4, 10, 10.1186/1754-9493-4-10 (2010). 20591192 PMC2902425

[b13] Abdelrazeq A. S. *et al.* Nicorandil-associated para-stomal ulceration: Case series. Eur. J. Gastroenterol. Hepatol. 18, 1293–1295, 10.1097/01.meg.0000243880.02197.8b (2006). 17099379

[b14] Toquero L., Briggs C. D., Bassuini M. M. & Rochester J. R. Anal ulceration associated with Nicorandil: case series and review of the literature. Colorectal Dis. 8, 717–720, 10.1111/j.1463-1318.2006.00972.x (2006). 16970585

[b15] Egred M., Andron M. & Morrison W. L. Nicorandil may be associated with gastrointestinal ulceration. BMJ 332, 889, 10.1136/bmj.332.7546.889 (2006). 16613962 PMC1440613

[b16] Campolmi N. *et al.* Corneal perforation: another side effect of nicorandil. Cutan. Ocul. Toxicol. 33, 96–98, 10.3109/15569527.2013.812105 (2014). 23845070

[b17] Fraunfelder F. W. & Fraunfelder F. T. Conjunctival and corneal ulceration associated with nicorandil. Cutan. Ocul. Toxicol. 33, 120–121, 10.3109/15569527.2013.811248 (2014). 23841868

[b18] Trechot F. *et al.* A case of nicorandil-induced unilateral corneal ulceration. Int Wound J 11, 238–239, 10.1111/iwj.12081 (2014). 23651162 PMC7950522

[b19] Smith V. M. & Lyon C. C. Nicorandil: do the dermatological and gastrointestinal risks outweigh the benefits? British Journal of Dermatology 167, 1048–1052 (2012). 23039135 10.1111/j.1365-2133.2012.11185.x

[b20] Hughes L. O., Rose E. L., Lahiri A. & Raftery E. B. Comparison of nicorandil and atenolol in stable angina pectoris. The American journal of cardiology 66, 679–682 (1990). 2144705 10.1016/0002-9149(90)91129-t

[b21] Di Somma S. *et al.* A double-blind comparison of nicorandil and metoprolol in stable effort angina pectoris. Cardiovascular drugs and therapy / sponsored by the International Society of Cardiovascular Pharmacotherapy 7, 119–123 (1993). 8485067 10.1007/BF00878320

[b22] Meeter K. *et al.* Efficacy of nicorandil versus propranolol in mild stable angina pectoris of effort: a long-term, double-blind, randomized study. Journal of cardiovascular pharmacology 20 Suppl 3, S59–66 (1992). 1282178

[b23] Lai C. *et al.* [A new anti-ischemic drug for the treatment of stable effort angina pectoris: nicorandil. Comparison with placebo and isosorbide-5-mononitrate]. Cardiologia 36, 703–711 (1991). 1839369

[b24] Zhu W. L. *et al.* Double-blind, multicenter, active-controlled, randomized clinical trial to assess the safety and efficacy of orally administered nicorandil in patients with stable angina pectoris in China. Circulation journal: official journal of the Japanese Circulation Society 71, 826–833 (2007). 17526976 10.1253/circj.71.826

[b25] Doring G. Antianginal and anti-ischemic efficacy of nicorandil in comparison with isosorbide-5-mononitrate and isosorbide dinitrate: results from two multicenter, double-blind, randomized studies with stable coronary heart disease patients. Journal of cardiovascular pharmacology 20 Suppl 3, S74–81 (1992). 1282180

[b26] Sasaki J. *et al.* A multicenter comparison of nicorandil and diltiazem on serum lipid, apolipoprotein, and lipoprotein levels in patients with ischemic heart disease. Cardiovascular drugs and therapy / sponsored by the International Society of Cardiovascular Pharmacotherapy 6, 471–474 (1992). 1450091 10.1007/BF00055603

[b27] Guermonprez J. L., Blin P. & Peterlongo F. A double-blind comparison of the long-term efficacy of a potassium channel opener and a calcium antagonist in stable angina pectoris. European heart journal 14 Suppl B, 30–34 (1993). 8370370 10.1093/eurheartj/14.suppl_b.30

[b28] Ulvenstam G. *et al.* Antianginal and anti-ischemic efficacy of nicorandil compared with nifedipine in patients with angina pectoris and coronary heart disease: a double-blind, randomized, multicenter study. Journal of cardiovascular pharmacology 20 Suppl 3, S67–73 (1992). 1282179 10.1097/00005344-199206203-00012

[b29] Swan Study, G. Comparison of the antiischaemic and antianginal effects of nicorandil and amlodipine in patients with symptomatic stable angina pectoris: the SWAN study. Journal of Clinical and Basic Cardiology 2, 213–217 (1999).

[b30] Chang C. H. *et al.* Risk of hospitalization for upper gastrointestinal adverse events associated with nonsteroidal anti-inflammatory drugs: a nationwide case-crossover study in Taiwan. Pharmacoepidemiol Drug Saf 20, 763–771, 10.1002/pds.2140 (2011). 21618340

[b31] Hippisley-Cox J., Coupland C. & Logan R. Risk of adverse gastrointestinal outcomes in patients taking cyclo-oxygenase-2 inhibitors or conventional non-steroidal anti-inflammatory drugs: population based nested case-control analysis. Bmj 331, 1310–1316, 10.1136/bmj.331.7528.1310 (2005). 16322018 PMC1298853

[b32] Hernandez-Diaz S. & Rodríguez L. A. G. Association between nonsteroidal anti-inflammatory drugs and upper gastrointestinal tract bleeding/perforation: an overview of epidemiologic studies published in the 1990s. Archives of Internal Medicine 160, 2093–2099 (2000). 10904451 10.1001/archinte.160.14.2093

[b33] Lee M. S., Lin R. Y., Chang Y. T. & Lai M. S. The risk of developing non-melanoma skin cancer, lymphoma and melanoma in patients with psoriasis in Taiwan: a 10-year, population-based cohort study. International journal of dermatology 51, 1454–1460, 10.1111/j.1365-4632.2011.05310.x (2012). 23171012

[b34] Chen Y. J., Wu C. Y., Shen J. L., Chen T. T. & Chang Y. T. Association between traditional systemic antipsoriatic drugs and tuberculosis risk in patients with psoriasis with or without psoriatic arthritis: results of a nationwide cohort study from Taiwan. Journal of the American Academy of Dermatology 69, 25–33, 10.1016/j.jaad.2012.12.966 (2013). 23375515

[b35] Chang C. H. *et al.* Association of thiazolidinediones with liver cancer and colorectal cancer in type 2 diabetes mellitus. Hepatology 55, 1462–1472, 10.1002/hep.25509 (2012). 22135104

[b36] Ray W. A. Evaluating medication effects outside of clinical trials: new-user designs. American journal of epidemiology 158, 915–920 (2003). 14585769 10.1093/aje/kwg231

[b37] Gagne J. J., Glynn R. J., Avorn J., Levin R. & Schneeweiss S. A combined comorbidity score predicted mortality in elderly patients better than existing scores. Journal of clinical epidemiology 64, 749–759 (2011). 21208778 10.1016/j.jclinepi.2010.10.004 PMC3100405

[b38] Parsons LS. Reducing bias in a propensity score matched-pair sample using Greedy matching techniques. In: Proceedings of the Twenty-Sixth Annual SAS Users Group International Conference, Long Beach, Calif., April 22–25, 2001. Cary, N.C.: SAS Institute, 2001.

[b39] Group I. S. Effect of nicorandil on coronary events in patients with stable angina: the Impact Of Nicorandil in Angina (IONA) randomised trial. Lancet 359, 1269–1275, 10.1016/S0140-6736(02)08265-X (2002). 11965271

[b40] Masoudi F. A. *et al.* Most hospitalized older persons do not meet the enrollment criteria for clinical trials in heart failure. American heart journal 146, 250–257 (2003). 12891192 10.1016/S0002-8703(03)00189-3

[b41] Hutchins L. F., Unger J. M., Crowley J. J., Coltman C. A. Jr. & Albain K. S. Underrepresentation of patients 65 years of age or older in cancer-treatment trials. The New England journal of medicine 341, 2061–2067, 10.1056/NEJM199912303412706 (1999). 10615079

[b42] Kapoor S. Nicorandil and its associated gastrointestinal side effects. Pacing and clinical electrophysiology: PACE 36, 662, 10.1111/pace.12105 (2013). 23437973

[b43] Smith V. M. & Lyon C. C. Nicorandil: do the dermatological and gastrointestinal risks outweigh the benefits? The British journal of dermatology 167, 1048–1052, 10.1111/j.1365-2133.2012.11185.x (2012). 23039135

[b44] King P. M., Suttie S. A., Jansen J. O. & Watson A. J. Perforation of the terminal ileum: a possible complication of nicorandil therapy. The surgeon: journal of the Royal Colleges of Surgeons of Edinburgh and Ireland 2, 56–57 (2004). 15570809 10.1016/s1479-666x(04)80140-9

[b45] Trechot P. *et al.* Role of nicotinic acid and nicotinamide in nicorandil-induced ulcerations: from hypothesis to demonstration. International wound journal 10.1111/iwj.12147 (2013). PMC7951002 24028540

[b46] Nomura A., Stemmermann G. N., Chyou P.-H., Perez-Perez G. I. & Blaser M. J. Helicobacter pylori infection and the risk for duodenal and gastric ulceration. Annals of Internal Medicine 120, 977–981 (1994). 7741826 10.7326/0003-4819-120-12-199406150-00001

[b47] Sebastian M., Chandran V., Elashaal Y. & Sim A. Helicobacter pylori infection in perforated peptic ulcer disease. British journal of surgery 82, 360–362 (1995). 7796009 10.1002/bjs.1800820325

[b48] Ng E. K. *et al.* Eradication of Helicobacter pylori prevents recurrence of ulcer after simple closure of duodenal ulcer perforation: randomized controlled trial. Annals of surgery 231, 153 (2000). 10674604 10.1097/00000658-200002000-00001 PMC1420980

[b49] Friedman G. D., Siegelaub A. & Seltzer C. C. Cigarettes, alcohol, coffee and peptic ulcer. New England journal of medicine 290, 469–473 (1974). 4810814 10.1056/NEJM197402282900901

[b50] Andersen I. B., Jørgensen T., Bonnevie O., Grønbæk M. & Sørensen T. I. Smoking and alcohol intake as risk factors for bleeding and perforated peptic ulcers: a population-based cohort study. Epidemiology 11, 434–439 (2000). 10874551 10.1097/00001648-200007000-00012

