# Supplementary material for: Use of nicorandil is Associated with Increased Risk for Gastrointestinal Ulceration and Perforation- A Nationally Representative Population-based study
Source: Sci Rep. 2015 Jun 29;5:11495. doi: 10.1038/srep11495 (PMC4483775; doi:10.1038/srep11495)
Supplement: Supplementary Information [file srep11495-s1.pdf]

Use of nicorandil is Associated with Increased Risk for Gastrointestinal Ulceration and Perforation- A Nationally Representative Population-based study

Chien-Chang Lee, Shy-Shin Chang, Shih-Hao Lee, Yueh-Sheng Chen, Wan-Ting Hsu, Meng-Tse Gabriel Lee.

Supplementary Information:

1. **Appendix 1.** Log (minus log) curves for the cumulative hazard for gastrointestinal ulceration (upper panel) and perforation (lower panel).
2. **Appendix 2.** Empirical predictor for prescription of Nicorandil and associated risk ratios of the propensity score model.
3. **Appendix 3:** Distribution of propensity score between nicorandil user and non-user.
4. **Appendix 4.** Receiver operating characteristic curve for the propensity score model.
5. **Appendix 5.** Participant Enrollment and Baseline Characteristics (PS matched)
6. **Appendix 6.** STROBE Statement.

Appendix 1. Log (minus log) curves for the cumulative hazard for gastrointestinal ulceration (upper panel) and perforation (lower panel).

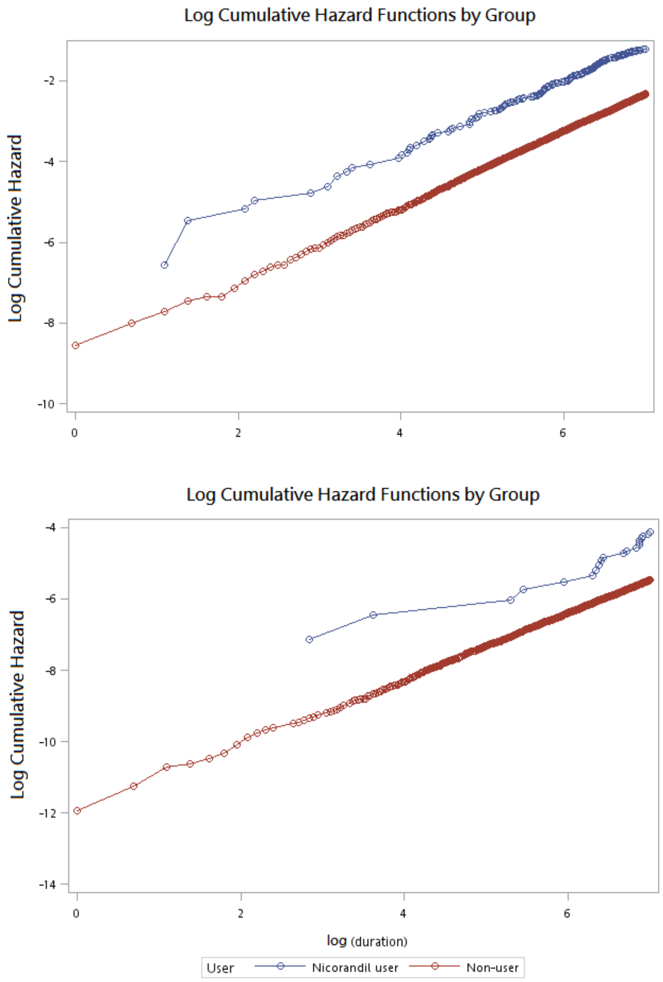

Appendix 2. Empirical predictor for prescription of Nicorandil and associated risk ratios of the propensity score model

Characteristics	Adjusted risk ratio (95%CI)	P-value
**Demographics**		
Gender male (%)	0.83 (0.70 - 0.98)	0.005
Age	1.35 (1.28 - 1.42)	<.0001
Age Squared	1.00 (1.00 – 1.00)	<.0001
Area (countryside area as reference)		
Urban Area	1.14 (0.87 – 1.49)	0.17
Metro Area	1.02 (0.78 – 1.33)	0.75
Suburban Area	1.01 (0.78 – 1.31)	0.66
Insurance type (dependent as reference)		
$1-$19,999	1.25 (0.92 – 1.69)	0.37
$20,000-$39,999	1.52 (1.11 – 2.07)	0.04
>=$40,000	1.64 (1.15 – 2.32)	0.01
**Comorbidity score**		
comorbidity score	0.98 (0.88 - 1.09)	0.73
**Baseline comorbidities**		
Diabetes	1.24 (0.92 - 1.68)	0.16
Alcohol/drug use	2.42 (1.05 - 5.60)	0.04
Tobacco Use	0.61 (0.20 - 1.82)	0.37
Psychiatric disorder	1.13 (0.91 - 1.40)	0.26
Neurologic disorder	1.26 (0.83 - 1.91)	0.28
Immunocompromised states	1.11 (0.83 - 1.48)	0.48
Cancer	0.82 (0.52 - 1.31)	0.41
Congenital renal disease	1.14 (0.16 - 8.39)	0.89
Acquired renal disease	0.96 (0.66 - 1.39)	0.81
Renal failure	1.80 (1.19 - 2.71)	0.01
Benign prostatic hyperplasia	1.34 (1.04 - 1.73)	0.03
Anemia	0.91 (0.60 - 1.38)	0.65
Spinal cord injury	1.91 (0.43 - 8.46)	0.39
Bed-ridden status	0.53 (0.26 - 1.08)	0.08
Obesity, diagnosed, not morbid	1.75 (0.71 - 4.34)	0.23
Malnutrition	0.79 (0.32 - 1.95)	0.60
Postgastric surgery	4.56 (0.52 - 40.0)	0.17
Hemodialysis	1.00 (0.13 - 7.79)	1.00
Chronic liver disease and cirrhosis	1.25 (0.97 - 1.61)	0.08
Serious neuromuscular	3.23 (0.83 - 12.5)	0.09
**Risk factors for Intestinal Perforation**		
appendicitis	0.81 (0.11 - 5.97)	0.84
Colorectal cancer	1.71 (0.75 - 3.90)	0.20
Stomach cancer (also called gastric cancer)	2.45 (0.56 - 10.7)	0.24
Inflammatory Bowel Disease (chronic)	1.01 (0.45 - 2.30)	0.98
Ulverative Enterocolitis	2.05 (0.27 - 15.3)	0.48
trauma (as exclusion for the intestinal perforation at the same time)	1.09 (0.83 - 1.44)	0.54
Crushing Injury	1.04 (0.38 - 2.84)	0.94

NSIAD	1.39 (1.12 - 1.73)	0.00
**Respiratory comorbidities**		
Chronic obstructive pulmonary disease (COPD)	1.13 (0.87 - 1.46)	0.36
Asthma	1.03 (0.75 - 1.40)	0.87
pulmonary heart disease	1.05 (0.32 - 3.41)	0.93
**cardiovascular comorbidities**		
Congestive heart failure	1.81 (1.32 - 2.50)	0.00
Cerebrovascular disease	1.10 (0.84 - 1.46)	0.49
Myocardial infarction/acute coronary syndromes	1.08 (0.67 - 1.76)	0.75
Stroke or transient ischemic attack	0.88 (0.60 - 1.28)	0.49
Peripheral arterial disease	1.21 (0.72 - 2.04)	0.47
Angina	1.86 (1.44 - 2.40)	<.0001
Other ischemic heart disease	2.31 (1.89 - 2.82)	<.0001
Cerebral atherosclerosis	1.25 (0.67 - 2.30)	0.49
Cardiac valve disease	0.82 (0.55 - 1.22)	0.33
Conduction disorder	0.93 (0.33 - 2.59)	0.88
Arrhythmia	1.19 (0.92 - 1.54)	0.19
Hypertension	1.36 (1.11 - 1.65)	0.00
Hyperlipidemia	1.01 (0.81 - 1.26)	0.94
**Percutaneous transluminal coronary angioplasty**		
PTCA	1.74 (1.02 - 2.97)	0.04
**Baseline musculoskeletal comorbidities**		
Ankylosing spondylitis	1.61 (0.78 - 3.32)	0.20
Gouty arthritis	1.14 (0.90 - 1.45)	0.28
Arthropathy associated with systemic disorders	1.19 (0.98 - 1.44)	0.08
**Risk factor**		
hypoproteinemia	1.41 (0.34 - 5.94)	0.64
Use of hydroxyurea	1.61 (0.21 - 12.5)	0.65
**Procedure**		
Intravenous infusion	0.96 (0.72 - 1.27)	0.77
Injection (broadly defined)	0.48 (0.07 - 3.46)	0.47
Complete blood count (or component) and sedimentation rate testing	1.12 (0.88 - 1.43)	0.36
CT	0.74 (0.55 - 1.01)	0.06
EKG tests	1.75 (1.41 - 2.18)	<.0001
Chest radiography	0.86 (0.64 - 1.16)	0.32
MRI	0.94 (0.77 - 1.14)	0.54
Blood gas analysis	0.86 (0.59 - 1.25)	0.42
Indwelling catheters	0.69 (0.48 – 1.00)	0.05
**Utilization**		
Number of OPD visit	1.01 (1.00 - 1.01)	<.0001
Number of emergency department visit	1.03 (1.00 - 1.06)	0.07
Number of hospitalization	0.93 (0.85 - 1.01)	0.07
**Medication**		
NSAIDs	1.03 (0.83 - 1.28)	0.77
Aspirin	1.90 (1.56 - 2.32)	<.0001
Systemic corticosteroids	1.10 (0.88 - 1.38)	0.39
DMARDs	0.89 (0.32 - 2.43)	0.82
Statin	1.24 (0.98 - 1.59)	0.08
ACE inhibitors	0.92 (0.75 - 1.13)	0.41
Oral hypoglycemic	1.07 (0.78 - 1.47)	0.69

Antipsychotic	0.2 (0.03 - 1.44)	0.11
Antidepressants	1.13 (0.87 - 1.46)	0.37

Appendix 3: Distribution of propensity score between nicorandil user and non-user

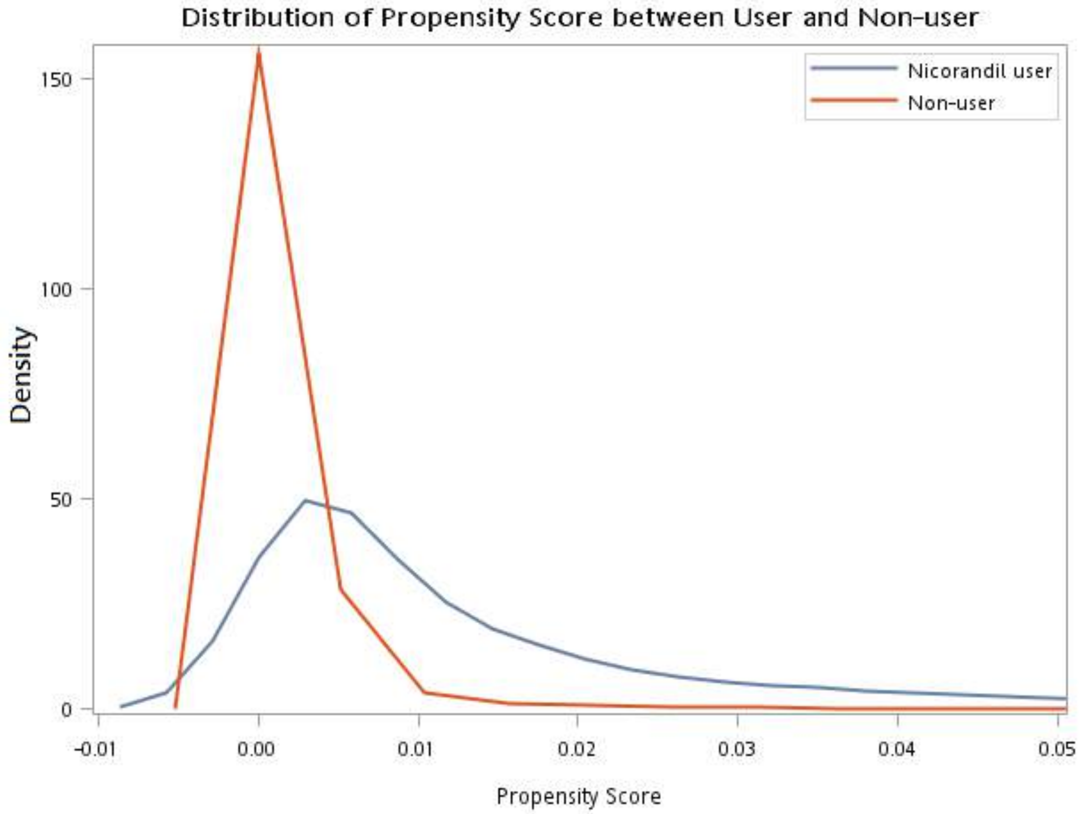

Appendix 4. Receiver operating characteristic curve for the propensity score model.

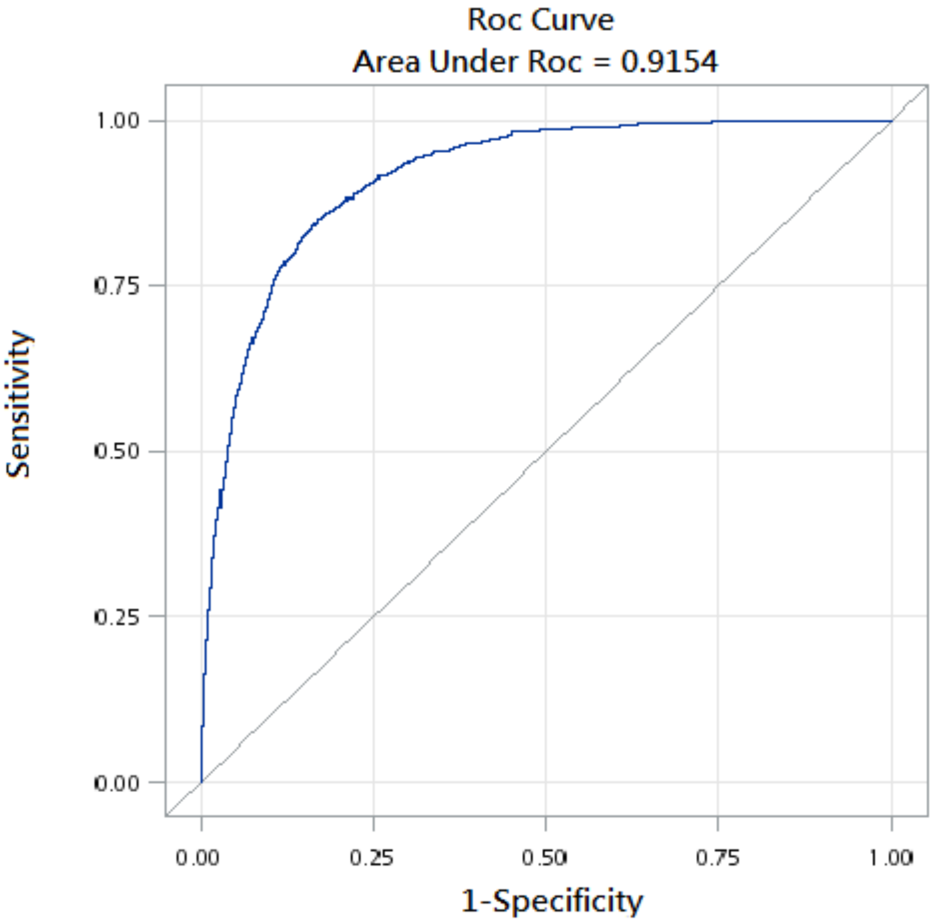

Appendix 5. Participant Enrollment and Baseline Characteristics (PS matched)

	**Cohort 1 : gastrointestinal ulcer**			**Cohort 2 : gastric perforation**		
	Nicorandil User (N=708)	Non-user (N=708)	Absolute Standardized Difference	Nicorandil User (N=1250)	Non-user (N=1250)	Absolute Standardized Difference
**Demographics**						
Gender male (%)	411 (58.0)	407 (57.5)	0.011	701 (56.1)	674 (53.9)	0.043
Age	65.1± 12.3	64.7 ± 11.5	0.008	65.9± 12.2	66.2 ± 11.3	0.006
**Area**						
Urban Area	212 (29.9)	224 (31.6)	0.036	357 (28.6)	389 (31.1)	0.055
Metro Area	195 (27.5)	190 (26.8)	0.015	346 (27.7)	336 (26.9)	0.017
Suburban Area	120 (31.1)	107 (29.2)	0.039	400 (32.0)	399 (31.9)	0.002
Countryside Area	81 (11.4)	87 (12.3)	0.026	147 (11.8)	126 (10.1)	0.053
**Insurance premium level**						
Dependent	55 (7.8)	59 (8.3)	0.020	105 (8.4)	126 (10.1)	0.058
$1-$19,999	211 (29.8)	181 (25.6)	0.094	390 (31.2)	391 (31.3)	0.001
$20,000-$39,999	306 (43.2)	313 (44.2)	0.019	555 (44.4)	547 (43.7)	0.012
>=$40,000	136 (19.2)	155 (21.9)	0.066	200 (16.0)	186 (14.9)	0.031
**Comorbidity score**						
Comorbidity score	0.52 ± 1.42	0.45 ± 1.37	0.017	0.65 ± 1.48	0.59 ± 1.48	0.014
**Baseline comorbidities**						
Diabetes	201 (28.4)	231 (32.6)	0.092	380 (30.4)	414 (33.1)	0.058
Disease related to use of alcohol	14 (2.0)	13 (1.8)	0.010	23 (1.8)	30 (2.4)	0.038
Disease related to use of tobacco	8 (1.1)	5 (0.7)	0.044	13 (1.0)	21 (1.7)	0.055
Psychiatric disorder	140 (19.8)	151 (21.3)	0.038	327 (26.2)	325 (26.0)	0.003
Neurologic disorder and spinal cord injury	28 (3.9)	33 (4.7)	0.034	58 (4.6)	65 (5.2)	0.025
Immunocompromised states	64 (9.0)	70 (9.9)	0.029	128 (10.2)	130 (10.4)	0.005
Cancer (excluding GI cancer)	32 (4.5)	40 (5.7)	0.051	66 (5.3)	61 (4.9)	0.018
Congenital renal disease and acquired renal disease	37 (5.2)	33 (4.7)	0.026	92 (7.4)	80 (6.4)	0.037
Renal failure and hemodialysis	42 (5.9)	39 (5.5)	0.018	93 (7.4)	73 (5.8)	0.064
Benign prostatic hyperplasia	87 (12.3)	81 (11.4)	0.026	176 (14.1)	178 (14.2)	0.004
Anemia	27 (3.8)	32 (4.5)	0.035	69 (5.5)	64 (5.1)	0.017
Bed-ridden status	11 (1.6)	12 (1.7)	0.011	25 (2.0)	34 (2.7)	0.047

Aortic dissection and aortic aneurysm	0	0	0	1 (0.1)	2 (0.2)	0.023
Obesity, diagnosed, not morbid	5 (0.7)	5 (0.7)	0	6 (0.5)	2 (0.2)	0.056
Malnutrition and postgastric surgery	6 (0.9)	4 (0.6)	0.034	14 (1.12)	11 (0.88)	0.024
Amputation	0	0		0	0	0
Chronic liver disease and cirrhosis	86 (12.1)	75 (10.6)	0.049	179 (14.3)	176 (14.1)	0.006
Organ transplant	0	0	0	1 (0.1)	2 (0.2)	0.023
Serious neuromuscular	3 (0.42)	5 (0.71)	0.038	4 (0.3)	6 (0.5)	0.025
**Gastrointestinal Risk factors**						
appendicitis	1 (0.1)	1 (0.1)	0	3 (0.2)	2 (0.2)	0.017
Colorectal cancer	8 (1.1)	12 (1.7)	0.047	14 (1.1)	14 (1.1)	0
Esophageal cancer	0	0	0	1 (0.1)	1 (0.1)	0
Stomach cancer (also called gastric cancer)	2 (0.3)	1 (0.1)	0.030	7 (0.6)	9 (0.7)	0.020
Inflammatory Bowel Disease (chronic)	6 (0.9)	5 (0.7)	0.015	16 (1.3)	18 (1.4)	0.013
Ulcerative Enterocolitis	1 (0.1)	2 (0.3)	0.030	1 (0.1)	0 (0.0)	0.040
superior mesenteric artery syndrome	0	0	0	0	0	0
trauma (as exclusion for the intestinal perforation at the same time)	58 (8.2)	67 (9.5)	0.044	119 (9.5)	127 (10.2)	0.021
Crushing Injury	4 (0.6)	5 (0.7)	0.018	11 (0.9)	8 (0.6)	0.027
ascariasis	0	0	0	1 (0.1)	2 (0.2)	0.023
Typhoid fever (acute)	0	0	0	0	0	0
**Respiratory comorbidities**						
Chronic obstructive pulmonary disease (COPD)	99 (13.9)	100 (14.1)	0.004	216 (17.3)	219 (17.5)	0.006
Asthma	56 (7.9)	55 (7.8)	0.005	115 (9.2)	110 (8.8)	0.013
Pulmonary heart disease	3 (0.4)	4 (0.6)	0.020	7 (0.6)	9 (0.72)	0.020
**Cardiovascular comorbidities**						
Congestive heart failure	96 (13.5)	92 (13.0)	0.016	179 (14.3)	153 (12.2)	0.061
Cerebrovascular disease	82 (11.5)	94 (13.3)	0.051	157 (12.6)	179 (14.3)	0.051
Myocardial infarction/acute coronary syndromes	22 (3.1)	13 (1.8)	0.081	45 (3.6)	40 (3.2)	0.022
Stroke or transient ischemic attack	39 (5.5)	57 (8.1)	0.101	72 (5.8)	73 (5.8)	0.003
Peripheral arterial disease	16 (2.3)	18 (2.5)	0.018	27 (2.2)	25 (2.0)	0.011
Angina	84 (11.8)	58 (8.2)	0.122	180 (14.4)	125 (10.0)	0.134
Other ischemic heart disease	237 (33.5)	222 (31.4)	0.045	448 (35.8)	416 (33.3)	0.053

Cerebral atherosclerosis	11 (1.6)	11 (1.6)	0	19 (1.5)	23 (1.8)	0.024
Cardiac valve disease	29 (4.1)	26 (3.7)	0.022	65 (5.2)	53 (4.2)	0.045
Conduction disorder	4 (0.6)	3 (0.4)	0.020	7 (0.6)	3 (0.2)	0.050
Arrhythmia	89 (12.6)	78 (11.0)	0.048	183 (14.6)	185 (14.8)	0.004
Hypertension	348 (49.2)	367 (51.8)	0.053	658 (52.6)	685 (54.8)	0.043
Hyperlipidemia	172 (24.3)	168 (23.7)	0.013	335 (26.8)	367 (29.4)	0.056
CV congenital anomalies (CA)	0	0	0	1 (0.1)	0	0.040
**Baseline musculoskeletal comorbidities**						
Ankylosing spondylitis	8 (1.1)	5 (0.7)	0.044	13 (1.0)	12 (0.9)	0.008
Congenital musculoskeletal anomalies	0	0	0	0	0	0
Gouty arthritis	91 (12.9)	90 (12.7)	0.004	172 (13.8)	169 (13.5)	0.006
Arthropathy associated with systemic disorders	252 (35.6)	273 (38.6)	0.061	501 (40.1)	507 (40.6)	0.009
**Healthcare Service Utilization**						
Number of OPD visit	25.8 ± 18.9	26.3 ± 20.1	0.009	30.6±22.1	30.9 ± 22.9	0.005
Number of emergency department visit	0.30 ± 0.82	0.30 ± 0.74	0	0.42±1.14	0.37 ± 0.96	0.016
Number of hospitalization	0.39 ± 0.91	0.35 ± 0.82	0.016	0.48±1.06	0.47 ± 1.10	0.003
**Medication**						
NSAIDs	317 (44.8)	324 (45.8)	0.019	627 (50.2)	630 (50.4)	0.004
Aspirin	295 (41.7)	280 (39.6)	0.043	523 (41.8)	509 (40.7)	0.022
Systemic immunosuppressive agents and biologics	0	0	0	1 (0.1)	3 (0.2)	0.040
Systemic corticosteroids	112 (15.8)	111 (15.7)	0.003	218 (17.4)	223 (17.8)	0.010
DMARDs	4 (0.6)	4 (0.6)	0	10 (0.8)	21 (1.7)	0.079
Statin	131 (18.5)	137 (19.4)	0.021	259 (20.7)	284 (22.7)	0.048
ACE inhibitors	146 (20.6)	151 (21.3)	0.017	275 (22.0)	299 (23.9)	0.045
Oral hypoglycemic	156 (22.0)	184 (26.0)	0.092	309 (24.7)	342 (27.4)	0.060
Antipsychotic	1 (0.1)	1 (0.1)	0	6 (0.5)	9 (0.7)	0.031
Antidepressants	81 (11.4)	77 (10.9)	0.017	204 (16.3)	215 (17.2)	0.023

Appendix 6. STROBE Statement—Checklist of items that should be included in reports of ***cohort studies***

	Item No	Recommendation
**Title and abstract** Everything addressed in pg1 and pg2	1	(*a*) Indicate the study's design with a commonly used term in the title or the abstract
		(*b*) Provide in the abstract an informative and balanced summary of what was done and what was found
Introduction		
Background/rationale addressed in pg3-4	2	Explain the scientific background and rationale for the investigation being reported
Objectives addressed in pg4	3	State specific objectives, including any prespecified hypotheses
**Methods**		
Study design addressed in pg4-9	4	Present key elements of study design early in the paper
Setting addressed in pg4-8	5	Describe the setting, locations, and relevant dates, including periods of recruitment, exposure, follow-up, and data collection
Participants addressed in pg4-7	6	(*a*) Give the eligibility criteria, and the sources and methods of case ascertainment and control selection. Give the rationale for the choice of cases and controls
		(*b*) For matched studies, give matching criteria and the number of controls per case
Variables addressed in pg4-9	7	Clearly define all outcomes, exposures, predictors, potential confounders, and effect modifiers. Give diagnostic criteria, if applicable
Data sources/ measurement addressed in pg4-9	8*	For each variable of interest, give sources of data and details of methods of assessment (measurement). Describe comparability of assessment methods if there is more than one group
Bias addressed in pg6-9	9	Describe any efforts to address potential sources of bias
Study size addressed in pg4-9	10	Explain how the study size was arrived at
Quantitative variables addressed in pg4-9	11	Explain how quantitative variables were handled in the analyses. If applicable, describe which groupings were chosen and why
Statistical methods addressed in pg6-9	12	(*a*) Describe all statistical methods, including those used to control for confounding
		(*b*) Describe any methods used to examine subgroups and interactions
		(*c*) Explain how missing data were addressed
		(*d*) If applicable, explain how matching of cases and controls was addressed
		(*e*) Describe any sensitivity analyses
**Results**		
Participants addressed in pg9-10	13*	(a) Report numbers of individuals at each stage of study—eg numbers potentially eligible, examined for eligibility, confirmed eligible, included in the study, completing follow-up, and analysed
		(b) Give reasons for non-participation at each stage
		(c) Consider use of a flow diagram
Descriptive data addressed in pg9-10	14*	(a) Give characteristics of study participants (eg demographic, clinical, social) and information on exposures and potential confounders
		(b) Indicate number of participants with missing data for each variable of interest
Outcome data	15*	Report numbers in each exposure category, or summary measures of

addressed in pg10-11		exposure
Main results<br>addressed in pg10-12	16	(*a*) Give unadjusted estimates and, if applicable, confounder-adjusted estimates and their precision (eg, 95% confidence interval). Make clear which confounders were adjusted for and why they were included
		(*b*) Report category boundaries when continuous variables were categorized
		(*c*) If relevant, consider translating estimates of relative risk into absolute risk for a meaningful time period

Other analyses addressed in pg11-12	17	Report other analyses done—eg analyses of subgroups and interactions, and sensitivity analyses
**Discussion**		
Key results addressed in pg12-13	18	Summarise key results with reference to study objectives
Limitations addressed in pg13-14	19	Discuss limitations of the study, taking into account sources of potential bias or imprecision. Discuss both direction and magnitude of any potential bias
Interpretation addressed in pg13-16	20	Give a cautious overall interpretation of results considering objectives, limitations, multiplicity of analyses, results from similar studies, and other relevant evidence
Generalisability addressed in pg14-15	21	Discuss the generalisability (external validity) of the study results
**Other information**		
Funding addressed in pg1	22	Give the source of funding and the role of the funders for the present study and, if applicable, for the original study on which the present article is based

*Give information separately for cases and controls.

Note: An Explanation and Elaboration article discusses each checklist item and gives methodological background and published examples of transparent reporting. The STROBE checklist is best used in conjunction with this article (freely available on the Web sites of PLoS Medicine at http://www.plosmedicine.org/, Annals of Internal Medicine at http://www.annals.org/, and Epidemiology at http://www.epidem.com/). Information on the STROBE Initiative is available at http://www.strobe-statement.org.
